# How does platform's fintech level affect its word of mouth from the perspective of user psychology?

**DOI:** 10.3389/fpsyg.2023.1085587

**Published:** 2023-02-16

**Authors:** Yongli Li, Xiaochen Ma, Yujia Li, Rui Li, Hongyu Liu

**Affiliations:** ^1^School of Economics and Management, Harbin Institute of Technology, Harbin, China; ^2^School of Business College, Changchun Guanghua University, Changchun, China

**Keywords:** fintech level, word-of-mouth, user experience, user trust, user stickiness, financial platform, structural equation model

## Abstract

**Introduction:**

The rapid development of fintech has brought opportunities for business operations and economic development. Currently, few researches have focused on how fintech level affects word-of-mouth (WOM) from the perspective of user psychology. Therefore, studying the effect of fintech level on WOM is a worthwhile scientific question.

**Methods:**

Based on motivation theory and reinforcement theory, this paper proposes a new psychology-based theoretical framework model to study the relationship between fintech level and WOM and constructs a structural equation model including fintech level, user experience, user trust, user stickiness and WOM through the analysis of 732 questionnaires.

**Results:**

The results indicate that the improvement of fintech level can enhance WOM. More specifically, fintech level has a significantly positive influence on user stickiness through two mediation variables (user experience and user trust), and further, user stickiness has a significantly positive influence on WOM.

**Discussion:**

This paper analyzes the internal mechanism of fintech level's influence on WOM from the micro psychological perspective, which enriches the psychology theoretical research. And, the conclusions provide specific suggestions for marketing and promotion of financial platforms in the future.

## 1. Introduction

The Financial Stability Board (FSB) provided the definition of financial technology (fintech), which is based on big data, cloud computing, artificial intelligence, blockchain, and a series of technical innovations, and applied to wealth management, retail banking, insurance, trading, settlement, etc. (Vives, 2017). The development of fintech brings new opportunities for social and economic growth and enterprise optimization management mode (Lagna and Ravishankar, 2022; Zhao and Zhou, 2022). Previous research has focused on how the application of fintech can improve the operational efficiency and profits of enterprises (Gomber et al., 2018) but ignored the impact of fintech applications on enterprise marketing, especially on user word of mouth (WOM). In fact, if a financial platform has a high fintech level (the quality of financial products and services provided by the fintech platform to users), it will be able to simplify consumer financial transactions, improve the efficiency of businesses' massive information processing, and quickly meet consumer needs. These advantages are probably to promote the WOM communication of the platform. Once the internal influence mechanism of the fintech level on WOM is found, it will be of great value to the marketing and promotion of the financial platform. To this end, this study aims to answer the following questions: (1) Whether the improvement of the fintech level can promote the WOM communication of financial platforms? (2) What factors play a significant mediation role in the process of the fintech level influencing WOM? (3) How should financial platforms develop their fintech to obtain a good WOM?

In terms of the relationship between fintech and WOM, some work has been explored in previous research. On the one hand, existing researchers have analyzed the relationship between fintech applications and WOM. Tian et al. (2019) found that the innovation and application of fintech not only brought more users to the financial platform but also reduced the security risk of the platform and facilitated the WOM of the platform. Roy et al. (2017) also found that the platform's fintech application increased user satisfaction and WOM in the retail industry. Similarly, Liu et al. (2022) found that electronic payment services, one of the significant fintech, could increase the perceived usefulness and payment efficiency of users, thereby increasing the WOM of financial platforms. On the other hand, existing research has explored the driving influence factors in the process of fintech's influence on WOM. Albayati et al. (2020) found that user trust played a vital mediation role in this process. To be specific, if fintech could be strictly regulated, it would bring users a sense of security and further promote the WOM communication of the platform. Lim et al. (2019) emphasized that in mobile fintech services, perceived usefulness played an important mediation role in the process of fintech's influence on WOM. In addition, Roy et al. (2014) focused on the positive impact of user loyalty in the fintech field on WOM.

According to the previous research progress, we determine that there is theoretical value in studying the relationship between the fintech level and WOM. However, there are weaknesses in the current research. First, although existing research has analyzed the relationship between fintech applications and WOM, most of them focused on analyzing the internal mechanism from the perspective of management theory, ignoring the impact of user psychology. Nevertheless, user psychology plays a crucial role in the process of WOM spread and cannot be ignored. Second, although a few research have paid attention to the psychological factors of individual users, the selection of research variables is not comprehensive enough. For example, Albayati et al. (2020) focused on the influence factors of user trust but ignored the role of user experience and other factors. Similarly, Lim et al. (2019) focused on the effect of perceived usefulness on WOM communication but did not focus on the influence of user trust. In fact, ignoring any one of the key factors may lead to bias in the research results and affect the scientific nature of the conclusion. In general, the lack of a psychological theoretical perspective and systematic research are the shortcomings of previous research.

To fill the gap, this study proposes a psychology-based theoretical framework model of the impact of the fintech level on WOM and six research hypotheses (see Section 2). It attempts to analyze the internal influence mechanism of the fintech level on WOM. Second, this study constructs a structural equation model (SEM) considering multiple factors, focusing on the analysis of the possible mediation effects in the process of the fintech level influencing WOM. Specifically, a questionnaire is designed to obtain user psychological data (including user experience, user trust, and user stickiness). Then an empirical analysis is conducted based on an SEM to systematically discuss the internal rules of the influence of the fintech level on WOM.

Compared with the existing research, our main work and contributions are listed below: (1) based on psychological theories, this study proposes a new theoretical framework for the influence of the fintech level on the platform's WOM, which improves the theoretical basis of the research on the relationship between fintech and WOM and enriches the research work in the field of WOM. (2) This study systematically analyzes and summarizes important influence factors in the process of the fintech level influencing WOM and includes them as mediation variables in the model to verify the two-level mediating effect and make up for the shortcomings of existing research. (3) To the best of our knowledge, this is the first time that researchers have taken Alipay, a famous fintech platform, as the research object and constructed an SEM to analyze the influence of the platform's fintech level on WOM, which provides a reference for subsequent research on the relationship between fintech level and WOM and provide management suggestions for financial platforms.

In order to clearly express the aforementioned work and contributions, the remainder of the study is organized as follows. Section 2 introduces the basic theories and proposes six relevant hypotheses in our research. Section 3 introduces the data sources and the measurement method of constructs in the SEM. Section 4 shows the results of the model assessment and hypothesis verification. Section 5 discusses the research and provides some suggestions. Section 6 provides the summary.

## 2. Theoretical analysis and research hypothesis

### 2.1. The influence of fintech level on user experience

Fintech could help us to develop various technologies for innovating financial products and services (Erel and Liebersohn, 2022). In terms of the fintech level, previous studies have focused on the level of fintech in various regions and interpreted the level of fintech as the fintech development status of a region (Yao et al., 2021; Wu et al., 2022). From a micro-perspective, some studies define the fintech level as the fintech development status of financial institutions such as banks (Cheng and Qu, 2020; Li et al., 2022). The fintech level proposed in our study refers to the fintech development status of a financial platform and also represents the fintech maturity of the financial platform. In fact, a user's perception of the fintech level of a financial platform mainly comes from the quality of its fintech products (Li et al., 2020; Di Maggio and Yao, 2021; Chen et al., 2022). Therefore, this study takes users' perception of fintech products (mobile payment, e-wallet, Alipay Huabei, robo-advisor, intelligent financial management, etc.) as the fintech level of the platform (Hendershott et al., 2021).

User experience is a significant concept in the psychology research field. The ISO 9241-210 standard provided a definition that user experience is people's impression of the product, system, or service that they are using or expect to use. That is, user experience is the sense of whether a financial product or service is convenient and useful (Filieri et al., 2015). Furthermore, in a study of the relationship between virtual reality and user experience, Shin (2021) defined user experience as immersion, which expresses the feelings of users in an interactive process. Similarly, user experience is the feelings and experiences obtained by users when using products or services, which includes both perceptual value and rational value (Park et al., 2018). According to previous research, we consider that user experience refers to the convenience of the Operating System (OS) and the aesthetics measure of the interface when users use a financial platform.

Many scholars have paid attention to the research on the relationship between fintech applications and user experience. For example, Lagna and Ravishankar (2022) affirmed that fintech could provide personalized services and a simple operation process to help low-income people make financial management and investment, which could improve user experience and develop inclusive finance. Nan et al. (2022) stated that face recognition payment (FRP) technology, one of the fintech, had relative advantages over traditional payment methods, which improved user experience and transaction efficiency. Similarly, Youn and Jin (2021) explained that the appearance of chatbots with artificial intelligence (AI) technology improved customer relationship management (CRM) and user experience. From the perspective of technology, Berman and Katona (2020) confirmed the adoption of AI algorithms on the financial platform could innovate specific products and services to satisfy users' preferences and improve user experience. It can be seen that previous research has demonstrated the positive impact of fintech applications on user experience from different research fields. In fact, fintech applications can be quantified in terms of the fintech level (the quality of financial products and services provided by the fintech platform to users). Therefore, we speculate that the fintech level also has a positive influence on user experience.

**Hypothesis 1 (H1):** The fintech level positively affects user experience.

### 2.2. The influence of user experience on user stickiness

As for the concept of user stickiness, many scholars have put forward their views from different aspects. On the one hand, Zott et al. (2000) considered that user stickiness is an important ability for enterprises to attract and retain customers. On the other hand, Lin et al. (2010) explained that stickiness is the time that customers spend on the company's social network. Furthermore, Roy et al. (2014) put forward a comprehensive viewpoint that stickiness includes two aspects, one is the visiting time of customers in the company's social network, and the other is the company's ability to retain customers. Therefore, in our study, user stickiness describes the long-term and high-frequency interactive attention of users, which reflects the loyalty of users to the platform.

Previous research has analyzed the relationship between user experience and user stickiness. Zhao and Zhou (2022) found that the user experience of the education platform had a significant and positive impact on the user's repurchase intention, and the user's repurchase intention will further affect user stickiness. In the research on willingness-to-use group purchase platforms, Wang et al. (2016) confirmed that user experience would positively affect user stickiness. In the retail business, Lian (2021) found that digitization retailers will improve user service experience and directly affect consumers' willingness to continue using. In addition, Brakus et al. (2009) revealed that consumers' evaluation of products was influenced by user experience, which would further affect consumers' brand loyalty (Japutra and Molinillo, 2019). From a theoretical point of view, Skinner proposed reinforcement theory, which is an important theory in the field of psychology. A reinforcement theory tells us that when a person takes an action, and the result is favorable to him, the action will be repeated in the future (Gordan and Krishanan, 2014). Therefore, it is reasonable to believe that when a user has a good experience on a platform, he has the motivation to continue using the platform, which increases the platform's user stickiness.

**Hypothesis 2 (H2):** User experience positively affects user stickiness.

### 2.3. The influence of fintech level on user stickiness

The aforementioned analysis has clearly claimed the concept of fintech level and user stickiness. Although there are few researchers who pay attention to their links, previous research has also confirmed the fact that technology has a significant effect on user stickiness. Vosooghi et al. (2019) claimed that the technology of robots had a positive influence on user stickiness because it could bring them good feelings. The adoption of AI-based chatbots increased user stickiness because it can bring convenience to users in the tourism business (Pillai and Sivathanu, 2020). In terms of mobile fintech, Lim et al. (2019) found that perceived security in mobile fintech services had a significant influence on users' confirmation, which further affected continual intention to use the services. The aforementioned researches illustrate one issue that technology or fintech may have a positive effect on user stickiness. In fact, this phenomenon can be explained by motivation theory. Self-serving is the motivation for self-enhancement (Berger and Milkman, 2012), which is a significant concept in psychology. We can imagine that improving the fintech level will better assist people in making wealth management and enabling users to make self-enhancement come true. Thus, there is a reason to believe that if a platform has a high fintech level, people will be willing to use the platform more frequently to achieve self-enhancement (Pillai and Sivathanu, 2020).

**Hypothesis 3 (H3):** The fintech level positively affects user stickiness.

### 2.4. The influence of fintech level on user trust

Trust can be understood as the confidence of one person in another, and it also can be defined as one's confidence in the reliability of an exchange partner (Morgan and Hunt, 1994). In our study, we focus on trust in fintech platforms (Bart et al., 2005; Zarifis and Cheng, 2022). User trust can also be interpreted as their confidence in the platform, which could bring them a sense of security and a stable income.

According to the previous research on the relationship between fintech level and user trust, Bunnell et al. (2020) proposed a recommendation system framework based on fintech. They verified the positive impact of the fintech application on improving user trust through empirical analysis. Albayati et al. (2020) affirmed that blockchain, one of the fintech, had the advantages of decentralization and asymmetric encryption, and its adoption would enhance users' sense of security and trust. Similarly, based on the constructed user trust evaluation model, Yan et al. (2020) found that with the support of big data mining, user trust can be improved. In fact, compared with manual data processing, mature fintech applications improves accuracy, security, and reliability. From a psychological point of view, establishing user trust is greatly influenced by technology security (Anderson, 2010). That is, if the platform's security can be improved, it will help to promote user trust. Therefore, we speculate that the higher the level of fintech, the more trust users will have in financial platforms.

**Hypothesis 4 (H4):** The fintech level positively affects user trust.

### 2.5. The influence of user trust on user stickiness

Previous research has focused on the relationship between user trust and user stickiness in many fields. User trust will effectively improve users' willingness and further improve user stickiness in the robot car rental industry (Vosooghi et al., 2019). In order to explore the influencing factors of the stickiness of member websites, El-Manstrly et al. (2020) found that user trust plays a more positive role than commitment. In addition, Lee and Hyun (2016) found that social networks and community participation had a positive impact on trust, and user trust further had a positive impact on user stickiness. In terms of the use of websites, user trust could motivate people to continue using the websites for a long time (Li et al., 2006). Also, knowledge and perceived security in mobile fintech services have a significant impact on user trust, and user trust positively affects users' intention to continue using the service (Lim et al., 2019). In fact, the theory of reasoned action (TRA) can explain this phenomenon. TRA tells us that people consider both the meaning and the consequences of an action before they do it (Kim et al., 2013). In the process, the individual's belief in the result of the behavior plays an important role, which will affect whether the individual takes the action. As far as this article is concerned, if users have trust in the financial platform, it means that users trust that financial products in the platform can bring them good profits, which may promote users to continue using the platform. Thus, we speculate that user trust has positive effects on user stickiness.

**Hypothesis 5 (H5):** User trust positively affects user stickiness.

### 2.6. The influence of user stickiness on WOM

The financial platform's online WOM, including consumer opinions and product reviews, is the main source of information for consumer purchasing decisions (Gu et al., 2012). WOM communication refers to informal interpersonal communication about a product, brand, or service, so it has been the main driving force for the website to acquire new members (Trusov et al., 2009; Baker et al., 2016). Therefore, users' comments and their intention to share the financial platform are uniformly summarized as the WOM of the platform in this study.

Some previous research studies use both user stickiness and WOM as the main indicators to measure user loyalty (Molinsimillo et al., 2020). However, we found that user stickiness and WOM do not belong to the same construct through the exploratory factor analysis, and user stickiness has a positive impact on WOM. In fact, user stickiness and WOM are different concepts (Roy et al., 2014). Specifically, user stickiness is an individual behavior, which mainly reflects the willingness of users to continue to use the platform (Kim et al., 2016). In comparison, owing to the good product quality of the platform, WOM emphasizes the willingness of users to promote the platform (Roy et al., 2017). Currently, a large number of studies have confirmed the positive impact of user stickiness on WOM (Roy et al., 2014; Kim et al., 2016). From the perspective of psychological theory, this phenomenon can be well explained by motivation theory. Specifically, altruistic motivation (Tellis et al., 2019) drives people to share valuable things with others. Because people share these things to show concern for others (Hennig-Thurau et al., 2004) and to try to help others (Lovett et al., 2013). In our research, if the platform's user stickiness is high, it indicates that users recognize the value of the platform, which motivates them to introduce the platform to those around them. Therefore, we propose the following final hypothesis.

**Hypothesis 6 (H6):** User stickiness positively affects WOM.

**Figure 1:**
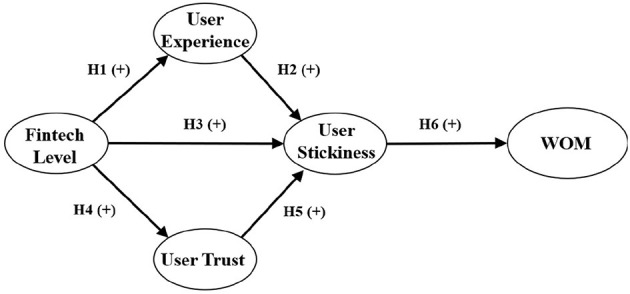
Theoretical framework.

According to the previous analysis, this study constructs an SEM that reflects the relationship between fintech level, user experience, user trust, user stickiness, and WOM (see Figure 1) to explore how the fintech level affects WOM and provide managerial suggestions.

## 3. Materials and methods

### 3.1. Participants

Alipay is a famous fintech platform, which is widely used in China to provide consumers with convenient fintech products and services. This study collects data on the fintech level, user experience, user trust, user stickiness, and WOM on Alipay through the survey. The specific workings are as follows in two stages. In the first stage, we make an online survey distributed to 107 users by the initial scales with 37 items first. Moreover, we used the exploratory factor analysis (EFA) to purify the original 37 items and the remaining 25 items in our scales, which eliminated the items with low loadings or high cross-loadings. In the second stage, we redesigned and reorganized the questionnaire in Questionnaire Star (a mainstream platform for academic surveys), distributing them through WeChat, QQ, and Alipay. Through this survey, we obtained 732 questionnaires (the total number of questionnaires is 766) after removing invalid questionnaires, with an effective recovery rate of 95.56%.

The details of the statistical results are as follows (see Table 1). First of all, 51.9% of the respondents were men, and 48.1% were women, with a gender ratio of nearly 1:1. The respondents are mainly aged between 18 and 39, accounting for 97.8%. The reason may be that people over 40 years old have little incentive to answer these questions on account of the infrequent use of fintech. Second, the education level of the respondents is mainly concentrated in the bachelor's degree (38.8%) and the master's degree or above (59.6%). Thus, it can be seen that the users of fintech are concentrated in people with high education. Finally, regarding the annual income of the respondents, the annual income of 50 thousand or less accounted for 32.2%, 50–100 thousand accounted for 21.3%, 100–200 thousand accounted for 33.3%, and 200 to 500 thousand accounted for 13.1%. The preliminary statistical analysis of the data indicates that the respondents have a balanced male-to-female ratio, high education level, stable income, etc. They could understand the meaning of the questionnaire design questions well and give accurate answers, which reflects the good representation of the sampled data in this study. A structural equation modeling methodology was used to determine how well the model fit the data and to identify support for each of the incorporated hypotheses.

**Table 1:** Demographic information of the survey sample group (*N* = 732).

**Measure**	**Category**	**Participants**	**Proportion** **(%)**
Gender	Male	380	51.91
	Female	352	48.09
Age	Under 18	0	0.00
	18–24	148	20.22
	25–39	568	77.60
	40–59	16	2.19
	60 or older	0	0.00
Education	High school graduated or below	12	1.64
	University bachelor's degree	284	38.80
	University master or doctoral degree	436	59.56
Annual income (individual)	Less than 50 thousand	236	32.2
	50–100 thousand	156	21.3
	100–200 thousand	244	33.3
	200–500 thousand	96	13.1
	More than 500 thousand	0	0.00

### 3.2. Measurement of constructs

The theorized model incorporates five constructs related to fintech level, user experience, users trust, user stickiness, and WOM. The items selected to measure the constructs are shown in Table 2 (for example, C1_Q1 represents the first item of the first construct. Similarly, C1_Q2 represents the second item of the first construct). In order to obtain data on respondents' degree of agreement toward each statement, we used 5-point Likert-type scales with “strongly disagree” and “strongly agree” as anchors (Wongkitrungrueng and Assarut, 2020). The following is an introduction to the specific measures.

**Table 2:** Main research measurement item properties.

**Construct**	**Label**	**Measurement Items^a^**	**Related Studies**	**Loading^b^**	**SMC**	**Cronbach alpha**
Fintech level	C1_Q1	Financial products pushed by big data and artificial intelligence (AI) have higher return rates when the risks are the same.	Setia et al., 2013; Lim et al., 2019; Pillai and Sivathanu, 2020; Yoon et al., 2022	0.507	0.257	0.810
	C1_Q2	Financial products pushed by big data and AI are timely.		0.677	0.458	
	C1_Q3	Financial products pushed by big data and AI cater to the user preference.		0.625	0.391	
	C1_Q4	There are plenty of financial products pushed by big data and AI.		0.691	0.477	
	C1_Q6	Big data and AI can adjust their behavior based on previous major political and economic events.		0.604	0.365	
	C1_Q6	Big data and AI improve your ability to handle financial business and manage money.		0.721	0.520	
User experience	C2_Q1	AI removes the space-time constraint (business can be handled online intelligently without manual intervention).	Lien et al., 2017; Park et al., 2018; Daragmeh et al., 2021	0.558	0.311	0.810
	C2_Q2	Fintech is easy to operate and use.		0.548	0.300	
	C2_Q3	Fintech page is user-friendly and beautiful.		0.770	0.593	
	C2_Q4	AI can understand your operations and commands.		0.641	0.411	
	C2_Q5	AI can automatically set and execute tasks based on your needs.		0.750	0.563	
	C2_Q6	AI can find your input error information and make a prompt.		0.560	0.314	
User trust	C3_Q1	You believe that the big data and AI can accurately judge the buying time of financial products.	Fang et al., 2014; Yang and Lin, 2014; Lim et al., 2019	0.717	0.514	0.851
	C3_Q2	You believe that the big data and AI have improved your return on investment.		0.893	0.797	
	C3_Q3	You believe that big data and AI have improved your investment security.		0.762	0.581	
	C3_Q4	You believe that the big data and AI will still bring earnings when the economy is down.		0.704	0.496	
User stickiness	C4_Q1	You are satisfied with the revenue from the platform fintech you are using (last year).	Hsu and Lin, 2016; Zhang et al., 2017; Ghali-Zinoubi and Toukabri, 2019	0.603	0.364	0.761
	C4_Q2	You are willing to continue using the platform's fintech products and services.		0.902	0.814	
	C4_Q3	You are willing to buy more fintech products and services.		0.812	0.659	
WOM	C5_Q1	Whenever there is a need for financial services, you will consider using this platform to query relevant informatio	Roy et al., 2014; Baker et al., 2016; Kim et al., 2016; Tellis et al., 2019	0.668	0.446	0.879
	C5_Q2	You are willing to tell others about this fintech platforms, products and services.		0.746	0.557	
	C5_Q3	You often mention this fintech platforms, products and services to your colleagues and acquaintances.		0.676	0.457	
	C5_Q4	You are willing to share details about this fintech platforms, products and services.		0.649	0.421	
	C5_Q5	You are willing to make positive comments about this fintech platforms, products and services.		0.822	0.676	
	C5_Q6	You are willing to recommend this fintech platforms, products and services to family members and friends.		0.827	0.684	

a Scale items were based on 5-point Likert-type scales. The scale ranged from 1 = “strongly disagree” to 5 = “strongly agree.”

b All loadings are standardized and significant at a *p*-value of < 0.001 levels.

#### 3.2.1. Fintech level

The fintech level is assessed with the development and application ability of fintech in the financial platform (Erel and Liebersohn, 2022). Especially, it refers to the ability of technology to help users invest and manage their money. Based on the statement of fintech, artificial intelligence, and other technologies in the existing literature (Hendershott et al., 2021; Shin, 2021; Zarifis and Cheng, 2022), we consider that the advantages of fintech are the progress than the traditional manual services, which can be summarized as two aspects. It can provide users with more personalized financial products and services through big data and artificial intelligence (Lagna and Ravishankar, 2022). Otherwise, products recommended by fintech have greater advantages in terms of security and reliability (Lim et al., 2019). Regarding the fintech level, this study proposes a scale with six items to measure the fintech level, “Financial products pushed by big data and AI are timely,” “Financial products pushed by big data and AI cater to the user preference,” etc.

#### 3.2.2. User experience

User experience is assessed with the users' feelings (Park et al., 2018). Based on the statement and scales of the user experience in the existing literature (Lee and Shin, 2018; Ghose et al., 2019), we consider that the core of user experience is to bring more convenient services to people and better meet the individual needs of users. The application of artificial intelligence (AI) technology optimizes the business process and reduces the time of user information review, which provides convenience for users (Youn and Jin, 2021). On the flip side, AI can understand the users' instructions and make a quick response, which meets the timely needs of users. Accordingly, we propose a scale with six items to obtain the data from respondents, “Fintech is easy to operate and use,” “AI can understand your operations and commands,” etc.

#### 3.2.3. User trust

User's trust is assessed with the trust of users in the technical capabilities of the platform, which not only includes the consideration of the security of fintech but also includes the trust in the fintech's capacity to bring users more considerable benefits (Zarifis and Cheng, 2022). Based on the previous analysis and existing literature (Bart et al., 2005; Wongkitrungrueng and Assarut, 2020), the previous trust scale is adapted to fit our research scenario. Thus, we propose a scale containing four items, such as “You believe that the big data and AI have improved your return on investment (ROI),” “You believe that big data and AI have improved your investment security,” etc.

#### 3.2.4. User stickiness

User stickiness is assessed by the user's loyalty to the financial platform (Brakus et al., 2009). That is, it is the user's willingness to continue using the platform. Previous researchers have made scales for constructs such as user intention to continue using (Wang et al., 2016), user loyalty (Lim et al., 2019), and user stickiness (Yoon et al., 2022) to explore the relationship between these constructs and the others. On this basis, we propose an evaluation scale for user stickiness to financial platforms, including three items in total, “You are willing to continue using the platform's fintech products and services,” “You are willing to buy more fintech products and services,” etc.

#### 3.2.5. Word of mouth

Word of mouth is the willingness of users to promote the financial platform. Previous research has focused on users' willingness to promote WOM on specific websites (Yu et al., 2017), mobile applications (Kim et al., 2016), and smart retail products (Roy et al., 2017) and proposed the scale of WOM measurement, respectively. With the existing results, we propose a quantitative standard containing six items, “You are willing to tell others about this fintech platforms, products and services,” “You are willing to share details about this fintech platforms, products and services,” “You are willing to recommend this fintech platforms, products and services to family members and friends,” etc.

## 4. Results

### 4.1. Measurement model assessment and analysis

First, the total 25 items' standardized loadings are greater than 0.5, and their related SMC is almost greater than 0.36 (see Table 2). This illustrates that our questions could represent their construct well and suggests convergent validity. Meanwhile, a confirmatory factor analysis (CFA) is also conducted to further validate the measures. After that, the results confirm all the measurement models have good fits in Table 3 (χ^2^/d.f. the range between 1.088 and 3.547, except for the fintech level; the goodness-of-fit index (GFI) range between 0.990 and 0.999; the adjusted goodness-of-fit index (AGFI) range between 0.948 and 0.990; normed fit index (NFI) range between 0.983 and 0.998; comparative fit index (CFI) range between 0.986 and 1.000; incremental fit index (IFI) range between 0.986 and 1.000; root-mean-square error of approximation (RMSEA) generally less than 0.08; root-mean-square residual (RMR) generally less than 0.05).

**Table 3:** Confirmatory factor analysis (a).

**Construct**	**Chi-square/d.f**.	**RMSEA**	**RMR**	**GFI**	**AGFI**	**NFI**	**CFI**	**IFI**
Fintech level	5.636	0.080	0.018	0.990	0.948	0.983	0.986	0.986
User experience	1.088	0.011	0.007	0.999	0.990	0.998	1.000	1.000
Users trust	1.871	0.035	0.010	0.997	0.987	0.997	0.999	0.999
User stickiness	-	-	-	-	-	-	-	-
WOM	3.547	0.059	0.008	0.995	0.968	0.995	0.997	0.997

-Values not available for scales containing only three items.

Second, the reliability of the measures is confirmed by several criteria. In our study, only one Cronbach's alpha value (user stickiness) is greater than 0.7, and all the others are greater than 0.8, which indicates that our scales are high reliabilities (see Table 2). Moreover, composite reliability (CR) ranges between 0.806 and 0.875 (see Table 4), which indicates that the items are consistent in one construct (Fornell and Larcker, 1981). In addition, the average variance extracted (AVE) for the constructs range between 0.411 and 0.612 in the measurement model (see Table 4), which also illustrates that our constructs have a good ability to explain their items.

**Table 4:** Confirmatory factor analysis (b).

**Construct**	**Mean**	**SD**	**Skewness**	**Kurtosis**	**CR**	**AVE**	**Fintech level**	**User experience**	**Users trust**	**User stickiness**	**WOM**
Fintech level	3.241	0.609	−0.977	2.647	0.806	0.411	**0.641** ^a^				
User experience	3.542	0.598	−0.451	1.626	0.807	0.415	0.550^**^	**0.644**			
Users trust	2.917	0.771	−0.297	0.687	0.854	0.597	0.554^**^	0.492^**^	**0.773**		
User stickiness	3.177	0.668	−0.380	0.799	0.822	0.612	0.480^**^	0.441^**^	0.572^**^	**0.782**	
WOM	3.202	0.671	−0.449	1.337	0.875	0.540	0.442^**^	0.460^**^	0.557^**^	0.648^**^	**0.735**

** Correlation is significant at the 0.01 level (two-tailed).

a Shaded numbers on the diagonal are the square root of AVE. Off-diagonal elements are correlations among different constructs. It shows that the diagonal elements are larger than the off-diagonal elements, which prove the discriminant validity.

Finally, our results also show strong evidence of validity. Convergent validity is indicated by the high loadings, composite reliability, and average variance extracted (Fornell and Larcker, 1981). Especially, there is an evaluation criterion that the model is good if composite reliability >0.7 and average variance extracted >0.36. The results of our data analysis are consistent with the aforementioned criteria (see Table 4). On the flip side, in order to illustrate the discriminant validity, we use the following criterion. The square root of AVE exceeds the other correlation coefficients in the same column or row (see Table 4). The aforementioned results show that our model has good discriminant validity. Thus, the results of the assessment described earlier (i.e., EFA and CFA) indicate the fact that the measurement model is reliable and valid. In addition, the mean value of each variable is approximately 3 (see Table 4). Meanwhile, the skewness of each variable is within ±1, and the kurtosis of each variable is within ±3, which proves that our variables obey the normal distribution (Kline, 2005).

### 4.2. Structural equation modeling results

The correlation matrix for the summary variables is presented in Table 4. The results show that the correlation coefficients are positive and significant at the 0.01 level among these constructs in the model, which is basically consistent with the results in Table 5.

**Table 5:** Parameter estimates and hypotheses tests.

	**Standardized**			
**Path**	**Hypothesis**	**Estimate**	***t*** **-value**	**Result**
Fintech level → User experience	H1	0.760	11.157	Supported
User experience → User stickiness	H2	0.327	4.463	Supported
Fintech level → Users stickiness	H3	0.112	1.366	Not supported
Fintech level → Users trust	H4	0.700	12.773	Supported
Users trust → User stickiness	H5	0.362	5.995	Supported
User stickiness → WOM	H6	0.842	12.450	Supported
Gender → WOM	-	0.015	0.553	Not supported
Age → WOM	-	0.074	2.544	Supported
Education → WOM	-	−0.020	−0.705	Not supported
Annual income → WOM	-	−0.011	−0.375	Not supported

-The relationship between control variables and dependent variables is not the focus of this research, and no hypothesis is proposed.

**Figure 2:**
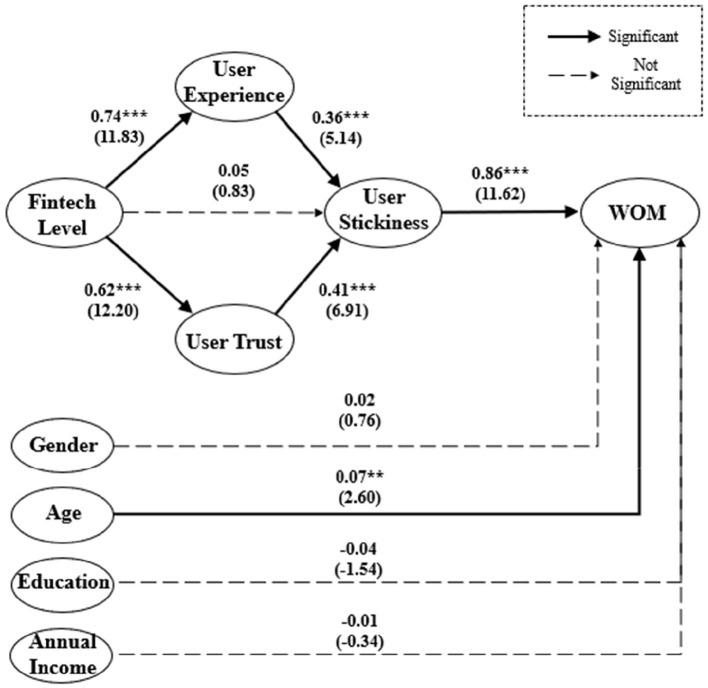
Structural equation model with standardized estimates (^***^significant at 0.001 level; ^**^significant at 0.01 level). χ^2^/d.f. = 4.022, *p* ≤ 0.001; GFI = 0.894; CFI = 0.903; IFI = 0.904, RMR = 0.036; RMSEA = 0.064.

In order to evaluate the results of the SEM accurately, we have done two studies. The first is to measure the reliability and validity of the SEM, and the second is to test the significance of constructs' coefficients in the SEM. In our research, Figure 2 shows the results of the structural equation modeling.

On the one hand, the data support the theoretical framework of the model, and the results show a good fit between the model and the observed data (χ^2^= 1323.30, degrees of freedom (d.f.) = 329, *p* ≤ 0.001, χ^2^/d.f. = 4.022; GFI = 0.894; CFI = 0.903; IFI = 0.904, RMR = 0.036; RMSEA = 0.064). Although a χ^2^/d.f. ratio of <2.0 is a common criterion to evaluate a good-fitting model, a statistically significant χ^2^/d.f. goodness-of-fit measure of >2.0 is not unusual with large sample sizes (Porter and Donthu, 2008). Therefore, we ought to use other factors to prove that our model is a good fit. Luckily, the value of RMSEA is lower than 0.08, and the value of RMR is lower than 0.05, which illustrates enough that our model is a good fit (Hu and Bentler, 1999).

On the other hand, the majority of hypotheses are supported, except for the relationship between the fintech level and user stickiness (see Figure 2). Specifically, the fintech level to user experience link (H1) is significantly positive, with a standardized estimate of 0.74 and a *t*-value of 11.83. The link from user experience to user stickiness (H2) is also significantly positive, with a standardized estimate of 0.36 and a *t*-value of 5.14. The fintech level to user trust link (H4) is significantly positive, with a standardized estimate of 0.62 and a *t*-value of 12.20. Also, user trust has a significantly positive influence on user stickiness, with a standardized estimate of 0.41 and a *t*-value of 6.91 (H5). Furthermore, the link from user stickiness to WOM (H6) is significantly positive, with a standardized estimate of 0.86 and a *t*-value of 11.62. Among the control variables, only one variable, age, plays a significant role. For these reasons, we consider that with the increase of age, people will accumulate more money and have more opportunities to make use of fintech to manage their wealth. Thus, they may easily get a deeper understanding of fintech's advantages and generate a good evaluation (WOM). Conversely, if people are younger and have fewer financial assets, they will have less opportunity and incentive to use fintech, which prevents WOM from being generated. Therefore, it is understandable that age as a control variable has a significantly positive effect on the WOM generation.

**Figure 3:**
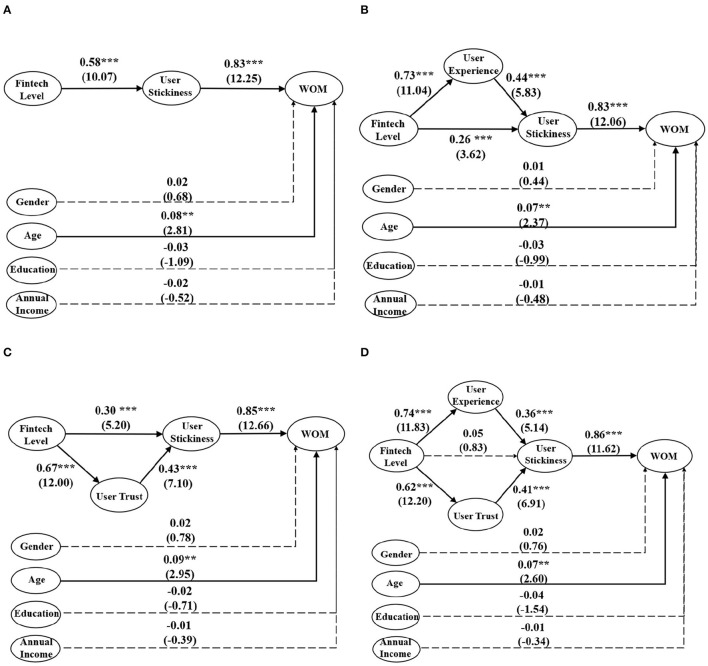
Test of mediation effect between fintech level and user stickiness (Solid arrows mean significant, dashed arrows mean insignificant; ^***^significant at 0.001 level; ^**^significant at 0.01 level). **(A)** No mediation effects (from fintech level to user experience). **(B)** User experience as the mediation. **(C)** User trust as the mediation. **(D)** Two mediation added (user experience and user trust).

However, the lack of support for the hypothesized link between the fintech level and user stickiness (H3) is surprising and unexpected, and this result leads us to rethink the model. To further discuss the relationship between the fintech level and user stickiness, we use stepwise regression (Baron and Kenny, 1986). First, we simplify the model to just three variables (fintech level, user stickiness, and WOM) and test the relationship among them. In Figure 3A, we find that the fintech level to user stickiness link (H3) is significantly positive with a standardized estimate of 0.58 and a *t*-value of 10.07. Second, we add user experience back into the SEM as a mediation variable from the fintech level to user experience. Through calculation, we find that user experience plays a good mediation effect. Meanwhile, the fintech level to user stickiness link (H3) is still significant, but the standardized coefficient decreases by 0.26 from 0.58 (see Figure 3B). Third, we remove user experience and add user trust to the model as mediation, and Figure 3C shows us a similar result with the second step. The result also shows user trust is a good mediation variable. The fintech level to user stickiness link (H3) is also significant, but the standardized coefficient decreases by 0.30 from 0.58 (see Figure 3C). In the end, we add both user experience and user trust to the model as mediation variables (see Figure 3D, it is exactly the same as Figure 2. This is shown again in Figure 3 for ease of reading). At this time, the result is the same as the previous analysis of Figure 2, the fintech level to user stickiness link (H3) is not significant. This phenomenon illustrates that user experience and user trust jointly play a complete mediation effect, and when either is absent, the mediation effect turns into a partial mediation effect. In our research, there are only two traces from the fintech level to user stickiness, and it must go through two mediation variables (user experience and user trust). It shows that the fintech level cannot directly increase the willingness of users to use their platform again. Still, it can increase user stickiness by improving user experience and enhancing user trust.

## 5. Discussion

In our research, we want to understand whether the fintech level of the financial platform could promote the improvement of WOM of the fintech platform. What mediation variables play a key role in this process? How should financial platforms develop their fintech to obtain good WOM? First, we find that user stickiness mediates the relationship between the fintech level and WOM, which indicates the fintech level could positively affect WOM *via* user stickiness. Second, we find user experience and user trust mediate the relationship between the fintech level and user stickiness, which indicates that the fintech level could affect stickiness *via* user experience or user trust. When there is only one mediation variable in the model (from the fintech level and user stickiness), it acts as a partial mediation (see Figures 3B, C). If we add both user experience and user trust into the model, they jointly play a complete mediation effect (see Figure 3D). This result illustrates that the fintech level could not directly affect user stickiness but could affect it *via* user experience and user trust. The aforementioned empirical results tell us that developing fintech is the right way for financial platforms to expand their influence. Finally, we also find that only one control variable, user age, has a significantly positive influence on WOM. It is because, with the increasing age of users, their demand for income assets and financial products is also increasing, which allows them to have more exposure and experience the advantages of fintech and generate more positive WOM.

Through the aforementioned research results, we find that if the financial platform wants to form a good WOM, it can adopt two methods: one is to improve user experience, and the other is to enhance user trust. In order to improve user experience, a financial platform could use fintech to extend the functions of online financial services, facilitate users to conduct financial business, and save more time for users. Meanwhile, they could provide users with some electronic instructions on fintech, which can help users better use fintech and have a good experience on the platform. Then, they could further develop the algorithm design so that the platform can better understand the user's command and demand and provide a better communication experience for users. In addition, they could also hire a professional to design the user page. It is because a friendly and beautiful page will brighten people's hearts and provide them good user experience.

On the flip side, to enhance user trust, a financial platform should focus on the development of fintech on improving investment returns. It is because the core of user trust in financial platforms is their trust in their profitability. Fintech can only be trusted by users if it is better able to reward them with revenue. If they want to increase user trust, they also need to further develop the algorithmic design and use fintech to improve platform security and profitability. In addition, users are also more concerned about whether big data and artificial intelligence technology will bring security threats to their private data. In other words, if a financial platform wants to improve users' trust, it should construct data security systems that could maintain and ensure the security of users' private data. For example, they can make use of data classification to manage users' data and use differential privacy technology to realize data sharing and security.

Our research fills the gap of previous research. Existing researchers have studied the relationship between fintech and the platform's WOM from the perspective of management theory (Sheng, 2021; Daud et al., 2022). This study proposes a new psychology-based theoretical framework model to study the relationship between the fintech level and WOM, which better explains how the fintech level affects WOM from a psychological perspective. In addition, although a few researchers have focused on the relationship between the fintech level and WOM, they have not considered comprehensive influencing factors (Roy et al., 2014, 2017; Kim et al., 2016). This study constructs multiple-step multiple mediator models, including three significant mediation variables (user experience, user trust, and user stickiness). We add more influencing variables than previous studies, so our research is more systematic. In addition, we confirm that the user experience and user trust jointly play a complete mediation effect. When either is absent, the mediation effect turns into a partial mediation effect (from the fintech level to user stickiness). This finding is important because it provides a good explanation of the underlying mechanism of the influence of the fintech level on WOM. This result can also provide more reliable management advice for financial platforms.

By analyzing our research's innovation and contribution, we conclude our academic implications. First, from the perspective of user psychology, this study proposes a new theoretical framework for the influence of the fintech level on the platform's WOM, which improves the theoretical basis of the research on the relationship between fintech and WOM and enriches the research work in the field of WOM. Second, based on the previous research and theoretical analysis, this study puts forward the measurement items of fintech level, user experience, user trust, user stickiness, and WOM, which provide a reference for the subsequent marketing promotion research. Third, from the perspective of micro-psychology, this study proposes a number of influence paths from the fintech level to WOM and verifies the positive effects of user experience, user trust, and user stickiness on WOM. This study better explains how the improvement of the fintech level promotes the financial platform's WOM *via* several significant constructs, which make an in-depth analysis and explanation of the underlying theoretical mechanism.

In terms of practical implications, this study systematically analyzes and summarizes important influence factors in the process of the fintech level influencing WOM and includes them as mediation variables in the model to verify the two-level mediating effect and make up for the shortcomings of existing research. This study has practical significance for brand marketing of financial enterprises. For instance, we have confirmed that the fintech level can have a positive impact on user engagement by influencing user experience and user trust. In real scenarios, financial enterprises can focus on the two key issues of improving user experience and ensuring user privacy security in the process of fintech research and development. In addition, this study is the first time that researchers have taken Alipay, a famous fintech platform, as the research object and constructed an SEM to analyze the influence of the platform's fintech level on WOM, which proposes a scientific paradigm for the study of WOM in other financial enterprises and provides a reference for subsequent research on the relationship between the fintech level and WOM.

Our research links the fintech level, user experience, user trust, user stickiness, and WOM together, studies the interaction mechanism among them, and proposes management suggestions based on academic theories. It is worth noting that our study focuses on how fintech brings convenience to the lives of individual users and promotes WOM communication. In fact, financial platform applications will also serve small- and medium-sized enterprises, so the influences on small- and medium-sized enterprises remain to be further discussed. This is also the issue that we will continue to explore in depth in the next step. This research direction will also help to enrich the theories of interdisciplinary subjects and better reveal the role of fintech development on human society.

## 6. Conclusion

Based on motivation theory and reinforcement theory, this study constructs an SEM (including fintech level, user experience, user trust, user stickiness, and WOM) and analyzes 732 questionnaires. After that, this study finds that the fintech level has a significantly positive influence on WOM *via* user stickiness. Furthermore, we find that user experience and user trust are two important mediation variables, and they jointly play a complete mediation effect. When either is absent, the mediation effect turns into a partial mediation effect (from the fintech level to user stickiness). This illustrates that there are only two traces from the fintech level to user stickiness, and it must go through two mediation variables (user experience and user trust). The conclusion of this study explores the influence path between the fintech level and WOM, which could help us understand the internal mechanism of WOM formation and remind us that WOM on financial platforms can be promoted by improving the fintech level. The research results of this study enriched the application scenarios of psychology and management and also provided a practical basis for the marketing and promotion of financial platforms and enterprises. In addition, from the perspective of psychology, it also provided research ideas for the relationship research between fintech development and social psychology.

## Data availability statement

The original contributions presented in the study are included in the article/supplementary material, further inquiries can be directed to the corresponding author.

## Author contributions

YoL and XM contributed to the conceptualization, research design, data collection, analysis work, and writing the article. YuL contributed to the communication, review, editing, and supervision. RL and HL contributed to the literature collection, analysis, inspection, and correction work. All authors contributed to the article and approved the submitted version.
